# Antibody–Drug Conjugates (ADC) in HER2/neu-Positive Gynecologic Tumors

**DOI:** 10.3390/molecules28217389

**Published:** 2023-11-02

**Authors:** Blair McNamara, Michelle Greenman, Nicole Pebley, Levent Mutlu, Alessandro D. Santin

**Affiliations:** Department of Obstetrics, Gynecology and Reproductive Sciences, Yale University School of Medicine, New Haven, CT 06520, USA

**Keywords:** antibody–drug conjugate, gynecologic malignancy, HER2, ERBB2, chemotherapy, cervical cancer, ovarian cancer, endometrial cancer

## Abstract

Antibody–drug conjugates (ADCs) are a new class of targeted anti-cancer therapies that combine a monoclonal tumor-surface-receptor-targeting antibody with a highly cytotoxic molecule payload bonded through specifically designed cleavable or non-cleavable chemical linkers. One such tumor surface receptor is human epidermal growth factor 2 (HER2), which is of interest for the treatment of many gynecologic tumors. ADCs enable the targeted delivery of a variety of cytotoxic therapies to tumor cells while minimizing delivery to healthy tissues. This review summarizes the existing literature about HER2-targeting ADC therapies approved for use in gynecologic malignancies, relevant preclinical studies, strategies to address ADC resistance, and ongoing clinical trials.

## 1. Introduction

Gynecologic malignancies, a major contributor to worldwide morbidity and mortality, are increasing in incidence and prevalence [1]. Chemotherapy, along with cytoreductive surgery, are fundamental in the treatment of gynecologic malignancies, whether newly diagnosed, recurrent, or metastatic. It is common for patients with recurrence or progression to require several lines and combinations of chemotherapies. Historically, doublet platinum chemotherapy has been widely utilized in these cancers but is associated with significant side effects. The rates of platinum resistance are also high [2]. For these reasons, immunotherapy and targeted drugs are now gaining momentum [3,4,5,6]. Given that gynecologic cancers encompass a wide variety of histologic subtypes, each with a host of associated genetic mutations that drive oncogenesis, there is remarkable potential for the use of targeted therapies.

**Figure 1:**
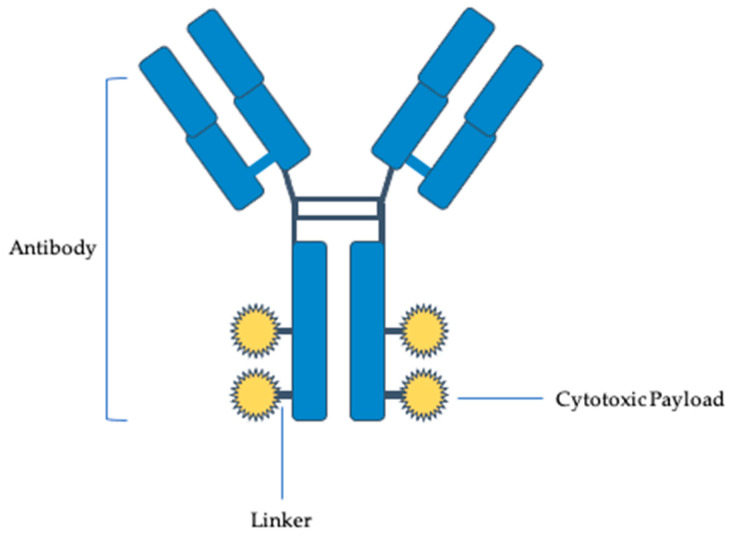
General structure of an antibody–drug conjugate.

**Figure 2:**
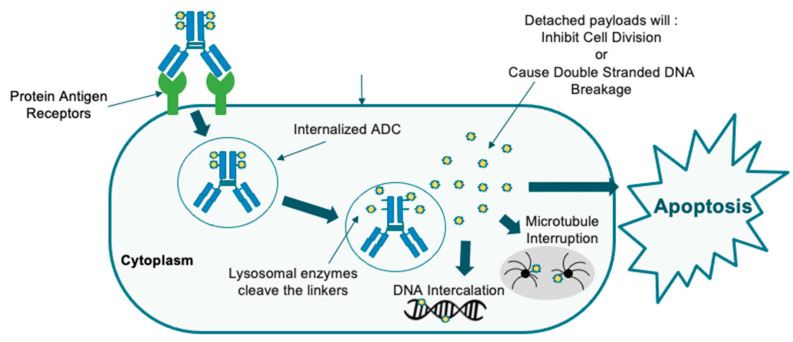
Core ADC mechanism of action.

**Figure 3:**
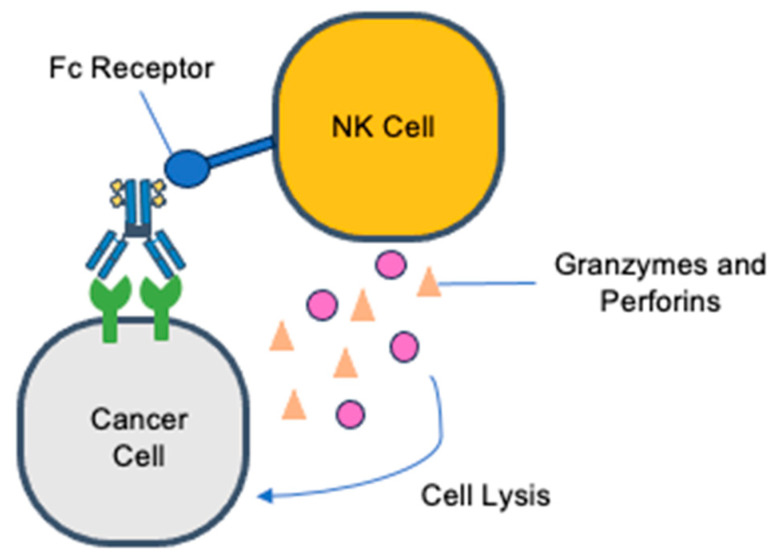
Fc-receptor-mediated antibody-dependent cellular cytotoxicity.

State-of-the-art targeted therapies are an exciting new frontier. Antibody drugs are the fastest-growing drug class, with many having been approved for therapeutic use and new antibody drugs in the pipeline [7]. Further antibody–drug conjugates (ADCs) are an opportunity to develop antibodies with novel mechanisms of action that can overcome conventional obstacles, with their efficiency rooted in the selection of the antibody, epitope, payload, linker, and drug-to-antibody ratio (DAR) [7]. ADCs have shown efficacy in other solid malignancies and are now being studied for their use in gynecologic cancers [8]. ADCs are composed of a humanized monoclonal antibody that is complementary to a specific tumor cell surface antigen, a cytotoxic payload, and a linker complex that connects the two (Figure 1). The payloads are either antimicrotubule compounds or DNA-damaging agents, which have cytotoxicity due to mitotic arrest and cell cycle death (Figure 2 and Figure 3) [9]. A fundamental component of ADCs is the controlled biodistribution of the payload to maximize drug delivery to tumor cells and minimize cytotoxicity elsewhere [10]. Noncleavable linkers have the advantage of greater stability in serum, while cleavable linkers have been observed to possess a greater bystander effect because of the high permeability of the payload into neighboring cells [11]. Whereas traditional platinum doublet chemotherapy causes cytotoxicity in off-target healthy cells, the paradigm-shifting technology of ADCs improves the specificity of drug delivery [8].

**Figure 4:**
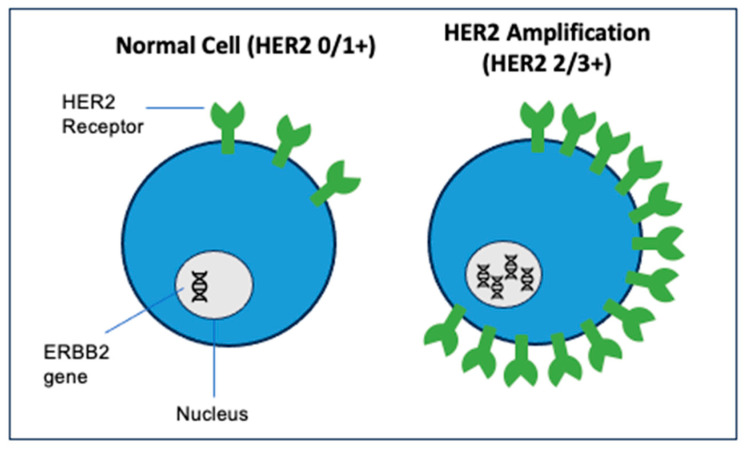
HER2 expression in normal cells vs. overexpression in malignant cells. (HER2 overexpression determined by immunohistochemistry (IHC) staining of 2+/3+ or fluorescence in situ hybridization assays (FISH) gene amplification).

ADCs are designed to target complexes in key cellular pathways that underly tumor proliferation and spread. One such pathway of interest in gynecologic cancers involves human epidermal growth factor receptor-2 (HER2). In gynecologic malignancies, HER2 is most often overexpressed in endometrial cancer (17–30%) and ovarian cancer (5–60%) [12,13,14]. HER2 is a tyrosine kinase receptor. The amplification of its parent gene, *ERBB2*, is seen in a range of solid malignancies (Figure 4). The dimerization of HER2 to other tyrosine kinases in the HER2 family is the initial step in the mitogen-activated protein kinase and PI3K signaling pathway [15]. This pathway ultimately leads to the transcription and translation of genes whose products bolster malignant cellular potential through an alteration of cell cycle regulation, protein transcription and translation, glucose metabolism, cellular migration, and cellular differentiation. HER2 overexpression is associated with chemotherapy resistance and unfavorable outcomes in both gynecologic malignancies and other solid tumors, making it an all-the-more valuable target for developing ADCs [13,16].

Although ADCs offer exciting potential, like all developing drugs, there are expected limitations to their use. As ADCs become increasingly utilized in the clinical setting, it is anticipated that resistance development will pose a threat, as seen with other anti-tumor agents [17]. Therefore, strategies to combat potential resistance, including careful selection of the target, the linker, and the payload, are critical to enhancing the efficacy of ADCs [17]. An additional consideration is the combination of ADCs with other agents known to be effective in tumors, such as immune checkpoint inhibitors, tyrosine kinase inhibitors, and chemotherapeutic drugs, to enhance ADC activity. Theoretically, ADCs are designed to have an enhanced safety profile via a stable linker to promote an improved therapeutic index; however, their successful implementation is often quite challenging [18]. Many of the undesired dose-limiting toxicities and adverse events seen with ADCs are similar to those of traditional anti-cancer therapy because the payloads use the same mechanism of action as chemotherapeutics and result in hematologic, neurologic, and hepatic toxicity [18,19]. As previously emphasized, a stable linker is required to minimize the non-specific systemic release of the cytotoxic drug from the monoclonal antibody in circulation [20].

While targeted therapies such as ADCs are widely recognized as the future of cancer treatment and already linchpins in the management of other solid malignancies, there is a dire need for trials demonstrating their safety and efficacy in gynecologic malignancies. There are only two Food and Drug Administration (FDA)-approved ADCs for the treatment of gynecologic malignancies at the time of writing: one on an ADC that targets a folate receptor subclass (Mirvetuximab soravtansine), and another that targets the tissue factor protein (Tisotumab vedotin) [21,22]. While there are not yet any FDA-approved HER2-targeting ADCs for gynecologic malignancies at present, several ongoing preclinical and clinical trials evaluating their safety and efficacy show significant promise and are reviewed here.

## 2. HER2 ADCs in Clinical Trials

### 2.1. Trastuzumab Deruxtecan

Trastuzumab deruxtecan (T-DXd, or DS-8201a) is a novel HER2-directed antibody–drug conjugate with established use in other solid tumors, currently under investigation for use in gynecologic malignancies. T-DXd consists of trastuzumab, a humanized monoclonal antibody, which targets the extracellular domain of HER2 cell surface receptors, a topoisomerase I inhibitor, and a linker protein (maleimide glycine-phenylalanine-glycine (GGFG) peptide). The tetrapeptide GGFG linker protein use is intentional, designed to be cleaved by tumors overexpressing lysosomal enzymes such as cathepsins B and L [23]. Following the internalization of T-DXd into tumor cells, DXd is liberated from the complex. DXd then infiltrates through the cell membrane to adjacent tumor cells causing DNA damage and cell death, independent of their HER2 status; therefore T-DXd exhibits clinical efficacy amongst heterogenous HER2 tumors [24]. The high membrane permeability of DXd allows for a significant bystander effect on cells expressing low HER2, which is an advantageous feature of the ADC T-DXd compared to the conventional monoclonal antibody alone [25].

The pharmacokinetics of T-DXd have been evaluated in multiple studies to understand the efficacy of the ADC. Utilizing xenograft mouse models and phosphor-integrated dots imaging, it has been demonstrated that the accumulation of T-DXd is correlated with the level of HER2, with no accumulation of DXd in HER2-negative tumors [25]. This thereby suggests that T-DXd binds to HER2-positive cells, then DXd acts on the surrounding cells through cell membrane permeability, independent of HER2 status [25]. It is likely that the effectiveness of T-DXd is reliant on HER2 protein expression, but unlike other HER2-targeted therapies, it does not require HER2 amplification [26]. Amongst cases of gastric and breast cancer with low or heterogenous HER2 expression, studies suggest T-DXd to be an effective treatment [27]. Additional factors contributing to the effectiveness of T-DXd include a higher drug-to-antibody ratio, facilitating increased delivery of the payload to target cells, high stability in plasma following intravenous infusion, and retained responsiveness [24,27].

Based on the approved FDA usage of T-DXd in metastatic breast cancer, non-small-cell lung cancer, and gastric cancer, the median half-life of T-DXd ranges from 5.4 to 6.1 days [28]. The relatively short half-life, consisting of days compared to weeks in their unconjugated components, adds to the stability of ADCs [29]. The primary metabolism of DXd is via the CYP3A4 system [28]. Utilizing radiolabeled DS-8201a, it was concluded that the major route of excretion for DXd is fecally, with the only detectable metabolite in urine and feces being unmetabolized DXd [30].

Based on completed clinical trials to date on T-DXd, the most common adverse effects are gastrointestinal and hematologic toxicities [31]. An FDA black-box warning for interstitial lung disease and pneumonitis has been reported with use of T-Dxd [28]. An additional warning is present for the risk of development of left ventricular dysfunction as a possible severe complication [28]. Due to special interest in these possible severe adverse effects, close monitoring with dose modification and discontinuation with signs of occurrence is recommended [32]. Overall, its safety profile is similar to that of related agents, and most oncology teams have become skilled at proactively preventing, monitoring, and managing its adverse events to facilitate the use of T-DXd safely [33].

T-DXd is now undergoing investigation in multiple clinical trials, specifically for its use in gynecologic malignancies [34]. Completed early-phase clinical trials have shown preliminary success with the use of trialed T-Dxd as well. The STATICE trial was a multicenter phase II, single-arm study amongst several centers in Japan evaluating the use of T-DXd. The enrollment eligibility required patients to have advanced or recurrent uterine carcinosarcomas (UCS) that had received prior chemotherapy. Various levels of HER2 expression were accepted [35]. The study recruited 32 patients, who were then stratified into cohorts based on HER2 expression, with 22 HER-2-high and 10 HER2-low-expressing tumors. The trial treatment regimen involved T-DXd administered as two doses (6.4 or 5.3 mg/kg) intravenously every 3 weeks with continued therapy until proven disease progression, unacceptable adverse effects, or withdrawal from the trial. Within the patients stratified as HER2-high, there was a reported response rate (ORR) of 54.5% (95% CI, 32.2 to 75.6), and amongst the HER2-low group, a rate of 70% (95%CI, 45.1 to 86.1). The median duration of response (DoR) was 6.9 months and 8.1 months for the HER2-high and HER2-low groups, respectively. Most notably, both groups had a 100% disease control rate regardless of HER-2 stratification.

Clinical trials have also demonstrated the effective use of T-DXd more broadly in patients with solid tumors, including endometrial cancer, such as in the Phase I trial (NCT02564900) conducted in the United States. Amongst the 59 patients with HER2-positive solid tumors enrolled in the trial, 2 had endometrial cancer and 1 had cervical cancer. Similar to the results of the STATICE trial, the potential benefits appear to be on the order of months: the reported median DoR was 11.5 months (95% CI, 7.0 to not met) and the median PFS was 7.2 months (95% CI, 4.8 to 11.1) [36]. This trial highlights the greatest success of T-DXd for tumor shrinkage in HER2-expressing patients.

With promising phase I outcomes, a follow-up multicenter phase II open-label trial (DESTINY-PanTumor02, NCT04482309) was conducted for HER2-expressing solid tumors. In total, 267 patients with solid tumors, including bladder, biliary tract, cervical, endometrial, ovarian, and pancreatic cancers, were enrolled, with ongoing data collection in progress. Preliminary data were reported at the ASCO 2023 annual meeting, and when specifically looking at uterine cancers, 57.7% of patients had either a partial or complete response to treatment. Most significantly, patients with HER2 tumors with an IHC of 3+ had the greatest response to treatment at 84.6%, with a response rate of 47.1% seen in HER2 tumors with an IHC of 2+.

Although T-DXd has demonstrated effectiveness, like other targeted HER2 therapies, it is also subject to resistance. The DAISY trial, investigating the use of T-DXd in metastatic breast cancer, was the first to explore the possible mechanisms of resistance. Tumor biopsies were analyzed using whole-exome sequencing at baseline and during progression with the use of T-DXd [37]. Cells with mutations in the SLX4 gene, which regulates endonucleases and resistance to topoisomerase I, required higher quantities of DXd for killing, implying that SLX4 loss contributes to resistance towards the payload activity [37,38]. IHC was performed on HER2 at baseline and at the time of disease progression. The majority of patients (65%), showed a decreased in HER2 expression, indicated a potential secondary resistance mechanism [37].

Despite threats of resistance, the utility of T-DXd has been exemplified through its FDA-approved use in non-gynecologic malignancies, including metastatic breast cancer, HER2-positive gastric cancer, and HER2-mutated non-small-cell lung cancer [39,40,41]. The proven success of this ADC as a treatment in other solid tumors as well as the existing preclinical evidence for its use in gynecologic malignancies make it a promising therapeutic alternative in the pipeline. We anticipate that forthcoming results from current clinical trials will provoke further studies on T-DXd and promote progress towards its approved clinical use.

### 2.2. Trastuzumab Duocarmazine

Trastuzumab duocarmazine (SYD985) is an ADC that utilizes the prodrug *seco*-duocarmycin-hydroxybenzamide-azaindole (*seco*-DUBA) as the cytotoxic agent. SYD985 incorporates a duocarmazine linker, which allows Trastuzumab duocarmazine to have a unique cytotoxic profile via binding to the minor groove in DNA, causing selective alkylation of N3 adenine residues [42]. The cell-permeable seco-DUBA released by SYD985 leads to efficient cell killing with cytotoxic effects in low-HER-2-expressing cells (IHC 1+, 2+), demonstrating that toxin activity is not exclusively dependent on HER-2 targeting [43]. Compared with T-DM1, SYD985 has shown a magnitude of 3–50 fold more cytotoxicity in low-HER2-expressing cells [43]. Phase I studies on breast cancer demonstrate that pharmacokinetics of the ADC follow a monophasic log–linear, time-independent decline [44]. This study found the half-life to consist of 2–3 days for the conjugated antibody when at doses of 1.2 mg/kg or higher and substantially lower levels of the free toxin compared to the concentration of the conjugated antibody [44]. Plasma stability is rooted in the use of duocarmycin for linker-drug technology. The low plasma stability of the antibody and rapid clearance from the systemic circulation result in low systemic exposure and reduced off-target toxicity. Therefore, the increased half-life is driven by the linker duocarmycin degradation [42]. The stable linker is also critical in preventing hepatotoxicity from the liver metabolism of seco-DUBA by stabilizing the ADC, preventing plasma release, and preventing unintentional off-target effects [42].

Clinical-trial-derived data on breast cancer show the most common adverse events of trastuzumab duocarmazine to include conjunctivitis, stomatitis, fatigue, and anorexia [45,46]. Grade 3 and 4 adverse events were an uncommon occurrence, and overall, SYD985 is considered a safe drug [46].

Trastuzumab duocarmazine was tested on a population of patients with HER2-positive endometrial, urothelial, gastric, and breast cancers in a Phase 1 dose-expansion clinical trial. There were a total of 14 endometrial cancer patients. The regimen consisted of 1.2 mg/kg Trastuzumab duocarmazine administered once every 21 days. A partial disease response was seen in five of the fourteen patients with endometrial cancer (39%, 95% CI, 13.9 to 68.4) [44].

NCT04205630 is a phase II trial of 64 patients with HER2-expressing recurrent, advanced, or metastatic endometrial carcinoma (Table 1). The inclusion criteria required patients to have disease progression while receiving, or following, platinum-based chemotherapy as their first-line treatment and HER2 immunohistochemistry scores of 1+, 2+, or 3+. Patients that had received two or more lines of chemotherapy due to advanced or metastatic disease were excluded. The release of the results is pending, with an anticipated primary outcome of ORR and secondary outcome assessments of PFS, OS, and AEs.

The use of Trastuzumab duocarmazine in ovarian cancer has not yet been explored in clinical trials, but preclinical studies provide a basis that this ADC may also have a role in the treatment of epithelial ovarian carcinomas. When compared to T-DM1, SYD985 was found to have significantly more cytotoxicity against epithelial ovarian carcinoma cell lines both in vitro and in vivo, regardless of HER2 expression. The higher cytotoxicity potency of SYD985 compared to T-DM1 is believed to be from the higher toxicity of the duocarmycin payload compared to the maytansin payload. This study showed a potent bystander effect, which highlights its versatility for treating epithelial ovarian carcinomas with heterogenous HER2 tumor expression [47].

Trastuzumab duocarmazine is an ADC with early-phase clinical trials (Table 2) demonstrating its disease response. Preclinical studies on its use in HER2 epithelial ovarian carcinoma, uterine serous carcinoma, and uterine and ovarian carcinosarcomas show antitumor activity and are anticipated to be the subject of further investigation [47,48,49].

## 3. Preclinical Development

Because HER2-targeted ADCs hold significant promise for improving clinical outcomes, additional combinations of antibody and payload systems are in the stages of preclinical development. These ADCs under development possess unique conjugation techniques, altering conjugation sites, increased drug-to-antibody ratios, and are capable of the bystander effect (Table 3) [9].

Excellent preclinical data exist for the use of two ADCs, trastuzumab emtansine (T-DM1) and DHES0815, in USC and UCS; however, neither is currently under investigation in clinical trials.

Trastuzumab emtansine (T-DM1) is composed of the HER2-directed monoclonal antibody trastuzumab attached via a non-cleavable thioethyl linker to emtansine [50]. Typically, 3–4 molecules of DM1 are conjugated to trastuzumab [51]. T-DM1 binds to the extracellular domain IV of HER2, is internalized, then, following degradation, the toxic payload DM1 is released into the cytoplasm, leading to cell cycle arrest and apoptosis [52,53]. T-DM1 distinguished itself by being the first ADC to receive FDA approval for use in HER2-positive metastatic breast cancer and has since become a widely utilized therapeutic option in advanced and recurrent cases [54].

In a study on HER2-overexpressing USC, the inhibition of USC cancer cell proliferation following T-DM1 was demonstrated in vitro. Subsequent in vivo studies on xenograft USC mice models treated with T-DM1 exhibited slower tumor progression compared to models treated with trastuzumab alone [51]. The clinical activity of TDM-1 in a HER2-overexpressing (3+ by IHC), recurrent, chemotherapy-resistant USC has been shown in one case report, in which a patient received TDM-1 and went on to experience complete resolution of disease as well as prolonged systemic control [55].

In a different study, the use of T-DM1 in uterine and ovarian carcinosarcoma overexpressing HER2 was evaluated in vivo and in vitro. T-DM1 was found to be significantly more effective in causing cell death than trastuzumab amongst both uterine and ovarian carcinosarcoma cell lines with HER2 overexpression. In carcinosarcoma cell lines with low or negative HER2 expression, minimal apoptotic activity was observed. In mice treated with T-DM1, a complete gross response was observed with an undetectable tumor throughout the duration of the study period. Conversely, mice treated with trastuzumab died due to tumor burden or required euthanasia due to the extent of the tumor growth [52].

An additional ADC, DHES0815A, uniquely utilizes a non-trastuzumab HER2-specific humanized monoclonal antibody linked to a DNA monoalkylating agent, pyrrolo [2,1-*c*] [1,4] benzodiazepine monoamide. DHES0815A binds to an alternative domain of the HER2 extracellular domain compared with ADCs that utilize trastuzumab, which precludes it from competing with other HER2-targeted agents and could potentially allow it to be used in combination with other HER2-targeted treatments [56,57].

Preclinical studies on DHES0815A in vitro in the USC cell line show significant antitumor cytotoxicity compared to a nontargeted control ADC (*p* < 0.05). Unlike T-DXd, DHES0815A had no significant cell cytotoxicity in USC without HER2 expression. In vivo mouse xenograft USC models reflected the preliminary in vitro results, with slower tumor growth and increased survival advantage in the DHES0815A-treated mice compared to the control mice (*p* < 0.01) [56].

Although fewer studies have been conducted on HER2-targeted ADCs in ovarian cancer, preclinical studies have shown their successful use. Disitamab vedotin (RC48-ADC) is a HER2-directed ADC made of an anti-HER-2 monoclonal antibody, Disitamab, bound to monomethyl auristatin E (MMAE) via a valine–citrulline cleavable linker [58]. Disitamab vedotin utilizes MMAE to bind to HER2-expressing cancer cells, where the ADC undergoes endocytosis, at which time enzymatic digestion causes the release of toxins that cleave the linker to release MMAE [59]. The release of MMAE in cytoplasm allows it to bind to microtubulins, causing damage to their structure and ultimately leading to cell cycle arrest and apoptosis [59,60]. MMAE concentrations are significantly higher in tumor tissues compared to serum, indicating that MMAE is more favorably released in tumor tissue, resulting in high tumor cytotoxicity [59,60]. Additional advantages of Disitamab vedotin include bystander killing and the ability to overcome tumor HER-2 heterogeneity [60,61].

A preclinical study on Disitimab vedotin showed that in vitro ovarian tumor cell lines underwent viability assays with sensitivity to Disitamab vedotin and tolerance to the nontargeting control ADC. Ovarian tumor cell lines negative for HER2 expression showed effective cytotoxicity, likely due to the bystander effect. In vivo mouse xenograft models showed results ranging from significantly inhibited growth to complete regression, depending on the dose received. Further, the anti-tumor effects in mice with the use of RC48-ADC were compared to T-DM1 and chemotherapy (paclitaxel, paclitaxel + cisplatin) and showed significant efficacy with lower toxicity [62].

In another study on ovarian cancer therapy, an alternative ADC was composed of trastuzumab conjugated to the cytotoxic molecule SN-38. Three iterations of the ADC were created using different types of linkers; an ester bond in T-SN38A, a carbonate bond T-SN38B, and a PEG linker in T-SN38C [63]. The payload, SN-38, is the active metabolite of irinotecan, which has anti-proliferation effects on tumor cells [63]. SN-38 independently has a narrow therapeutic window, prohibiting it from being used alone as therapy; however, its delivery in the form of an ADC allows for its safer use [63,64]. The in vitro results showed a strong cytotoxic efficiency of T-SN38, which was not paralleled with trastuzumab alone. In vivo, all three T-SN38 groups and trastuzumab had an impact on tumor growth; however, a tumor regrew in the trastuzumab group during the study period. In both the in vitro and in vivo experiments, T-SN38B had the best therapeutic effect, proposed to be due to its longer half-life.

## 4. Overcoming Resistance

**Figure 5:**
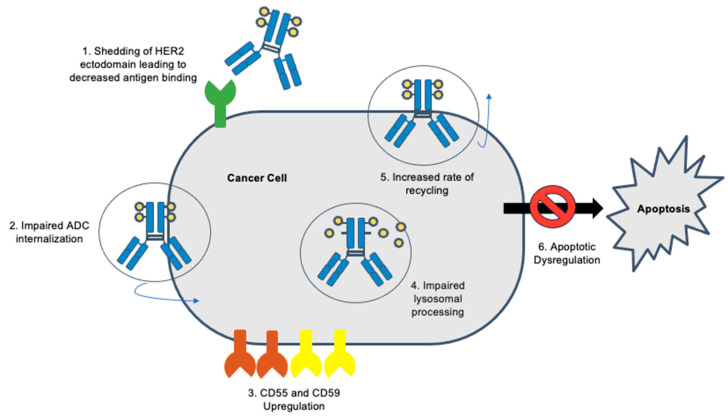
Possible mechanisms of resistance to HER2 ADCs.

At present, the mechanisms of resistance to ADCs directed against HER2 are poorly characterized, given that their use in gynecologic malignancies remains in the preclinical and clinical trial phases only. However, for the more prolific use of FDA-approved HER2-directed ADCs in breast cancer and other solid tumors, several mechanisms of resistance have been described. It can be extrapolated and anticipated that with the use of HER2 ADCs in gynecologic malignancies, resistance will develop as well. Common resistance mechanisms include antibody-mediated resistance with reduced target expression, impaired endocytosis preventing ADC internalization, disrupted lyososomal function, and payload resistance (Figure 5) [65].

Novel strategies are being explored to both prevent and overcome resistance to allow ADCs to remain a promising treatment option. The most appealing solutions involve combining anti-HER2 ADCs with cytotoxic agents with alternative modes of action such as immune checkpoint inhibitors, statins, tyrosine kinase inhibitors, and DNA-damaging agents [66]. By combining drugs, the agents can overcome developing resistance while taking caution to avoid overlapping toxicities [11].

Checkpoint inhibitors, which have independently proven their effectiveness in cancer therapy, have the ability to turn immunologically cold tumors into hot tumors with an anti-tumor T cell response [67]. ADCs induce strong immunogenic cell death due to the pro-inflammatory properties of the toxic payloads, making them an attractive option to be paired with immune checkpoint inhibitors [68]. The combination of a checkpoint inhibitor with an ADC would lead to the destruction of tumor cells, independent of the down-regulation or loss of antigen the ADC is directed towards [67]. Although the potential for synergistic effects is present in the sequential treatment of an ADC followed by a checkpoint inhibitor, further studies are required to determine the clinical impact this could have and to circumvent the adverse effects that occur when combining treatment modalities.

The use of statins in cancer treatment is not a new concept. For many years, it has been known that statins pose anticancer activity, varying from cell-cycle arrest to the induction of apoptosis to the inhibition of metastatic lesions, suggesting that they can effectively be combined with other therapies [69]. In gastric PDX models, the addition of a statin to T-DM1 increased HER2 membrane stability, allowing ADC binding to be increased and subsequently requiring lower doses of the ADC to achieve the same therapeutic effect [70]. Similarly, in breast cancer, statin use together with T-DM1 resulted in significantly increased overall survival (*p* = 0.016) [71]. Although statins modulate antitumor activity via multiple mechanisms, in this circumstance, it is believed that statins overcome anti-HER2 resistance by inhibiting downstream signaling when HER2 signaling is blocked [72].

While both ADCs and tyrosine kinase inhibitors (TKIs) target HER2, TKIs act on an intracellular domain while ADCs act extracellularly [73]. These different targets allow TKIs and ADCs to work differently, and therefore they can theoretically be given together to improve efficacy [73]. Tyrosine kinase inhibitors can widen the therapeutic index of an ADC by altering surface antigen availability to sensitize low-antigen-expressing tumor cells [11]. A meta-analysis on the addition of tyrosine kinase inhibitors to trastuzumab has shown promise in breast cancer trials [74]. It is proposed that in cases of breast cancer with trastuzumab resistance, the addition of a TKI aids in overcoming resistance due to the shedding of the extracellular domain of HER2, which typically results in less effective binding of the unconjugated antibody [74].

ADCs combined with chemotherapy have received FDA approval thus far for hematologic cancers and urothelial cancers [11]. The rationale for combining ADCs with chemotherapy is multi-faceted. The payload component of ADCs causes DNA damage, and when paired with chemotherapy, this inhibits DNA repair, resulting in cell cycle arrest, allowing for a synergistic effect. Further, chemotherapy paired with an ADC may modulate surface antigen expression, with upregulation allowing the ADC to work more effectively [11]. Thoughtful consideration of an ADC and chemotherapy combination is obligatory due to potentially overlapping toxicity profiles to avoid intolerable combinations.

## 5. Conclusions

The rates of gynecologic malignancies are increasing worldwide; the most commonly used chemotherapy regimens, platinum doublets, are associated with high rates of treatment-limiting side effects and platinum resistance. There is an imperative need for effective targeted therapies such as ADCs, which specifically target cells with cancerous changes and halt processes that enable malignant transformation and spread. HER2-directed ADCs in particular have the potential to target some of the rarest and most aggressive subtypes within gynecologic malignancies. With continued research interest and investment into trials such as those highlighted in this review, HER2-targeting ADCs may revolutionize the treatment of common gynecologic malignancies and confer not only a survival benefit when compared to traditional platinum chemotherapy but also dramatically improve quality of life.

## Figures and Tables

**Table 1:** Summary table of HER2-directed ADCs under development for gynecologic malignancies.

ADC	Target Antigen	Payload	Mechanism of Action	Gynecologic Cancer Type	Trial Phase	Clinicaltrials.gov Identifiers	Key Arms
Trastuzumab deruxtecan (DS-8201a)	HER2	Deruxtecan (Exatecan derivative)	inhibition of topoisomerase I	Endometrial, Ovarian, Cervical	II	NCT04482309	DS-8201a monotherapy
Trastuzumab Duocarmazine (SYD985)	HER2	Duocarmycin	DNA alkylation	Endometrial, Ovarian	II	NCT04205630	SYD985 monotherapy
					I	NCT04235101	SYD985 + Niraparib at various doses

**Table 2:** Summary of active * clinical trials testing HER2-targeting agents in solid tumors.

Clinicaltrials.gov Identifiers	Phase	Drug	Drug Class	Study Population
NCT02491099	2	Afatinib	Tyrosine Kinase Inhibitor	Persistent or recurrent HER2-positive USC
NCT04235101	1	Trastuzumab Duocarmazine (SYD985)	ADC	HER2-expressing recurrent, advanced or metastatic endometrial carcinoma
NCT04294628	1	Trastuzumab deruxtecan (T-DXd, DS-8201a)	ADC	HER2 expressing metastatic or unresectable solid tumor
NCT04482309	2	Trastuzumab deruxtecan (T-DXd, DS-8201a)	ADC	Locally advanced, metastatic, or unresectable endometrial cancer with HER2 overexpression (Cohort 4)
NCT04585958	1	T-DXd + olaparib	ADC	HER2-expressing metastatic/unresectable cancers, with dose expansion phase limited to endometrial serous carcinoma
NCT04639219	2	Trastuzumab deruxtecan (T-DXd, DS-8201a)	ADC	HER2-overexpressing metastatic or unresectable solid tumor
NCT04704661	1	Trastuzumab deruxetcan with ceralasertib		Advanced endometrial cancer with HER2-positive expressing with progression following at least one line of systemic chemotherapy
NCT05256225	2/3	Trastuzumab/pertuzumab or trastuzumab with carboplatin-paclitaxel	Monoclonal antibody	Primary, chemotherapy-naïve, HER2-positive endometrial serous carcinoma or carcinosarcoma
NCT05372614	1	Trastuzumab deruxtecan with neratinib	ADC + Kinase Inhibitor	HER2 expressing metastatic or unresectable solid tumor
NCT05765851	1	T-DXd + DS-1103a	ADC	HER2-positive metastatic or unresectable solid tumor

USC, uterine serous carcinoma. * Active based on information provided on ClinicalTrials.gov (accessed 30 August 2023).

**Table 3:** Summary of ADC components.

ADC	Monoclonal Antibody	Linker	Payload	DAR
Trastuzumab Deruxtecan (T-DXd)	Trastuzumab	Cleavable	Deruxtecan	8
Trastuzumab duocarmazine (SYD985)	Trastuzumab	Cleavable	Duocarmycin (Seco-DUBA)	2.8
Ado-trastuzumab Emtansine (T-DM1)	Trastuzumab	Non-cleavable	Emtansine (DM1)	3.5
DHES0815A	IgG antiHer2	Non-cleavable	Purrolo [2,1-c] [1,4] benzodiazepine monoamide (PBD-MA)	2
Disitamab Vedotin (RC-48)	Hertuzumab	Cleavable	Monomethyl auristatin E (MMAE)	4
Trastuzumab-SN-38 conjugates (T-SN38)	Trastuzumab	Cleavable	SN-38	A: 3.7 B: 3.2 C: 3.4

DAR = Drug Antibody Ratio.

## Data Availability

Not applicable.
